# The Association of Standardized Patient Educators (ASPE) Standards of Best Practice (SOBP)

**DOI:** 10.1186/s41077-017-0043-4

**Published:** 2017-06-27

**Authors:** Karen L. Lewis, Carrie A. Bohnert, Wendy L. Gammon, Henrike Hölzer, Lorraine Lyman, Cathy Smith, Tonya M. Thompson, Amelia Wallace, Gayle Gliva-McConvey

**Affiliations:** 1 Clinical Learning and Simulation Skills Center, The George Washington University School of Medicine and Health Sciences, Washington, DC USA; 2 Standardized Patient Program, University of Louisville School of Medicine, Louisville, KY USA; 3 New England Clinical Skills Consulting, Westborough, MA USA; 4 Medizinische Hochschule Brandenburg Theodor Fontane, Neuruppin, Germany; 5 Sentara Center for Simulation and Immersive Learning, Eastern Virginia Medical School, Norfolk, VA USA; 6 Division of Training and Simulation, The Centre for Education and Knowledge Exchange in Aging, Baycrest Health Sciences, Toronto, Ontario Canada; 7 Simulation and Education Center, Arkansas Children’s Hospital, University of Arkansas for Medical Sciences, Little Rock, AR USA

**Keywords:** Patient simulation, Simulation training, Standards, Simulated patient, Standardized patient, Simulated patient methodology, Standardized patient methodology, Case design, Feedback, Training

## Abstract

In this paper, we define the Association of Standardized Patient Educators (ASPE) Standards of Best Practice (SOBP) for those working with human role players who interact with learners in a wide range of experiential learning and assessment contexts. These human role players are variously described by such terms as standardized/simulated patients or simulated participants (SP or SPs). ASPE is a global organization whose mission is to share advances in SP-based pedagogy, assessment, research, and scholarship as well as support the professional development of its members. The SOBP are intended to be used in conjunction with the International Nursing Association for Clinical Simulation and Learning (INACSL) Standards of Best Practice: Simulation^SM^, which address broader simulation practices. We begin by providing a rationale for the creation of the ASPE SOBP, noting that with the increasing use of simulation in healthcare training, it is incumbent on ASPE to establish SOBP that ensure the growth, integrity, and safe application of SP-based educational endeavors. We then describe the three and a half year process through which these standards were developed by a consensus of international experts in the field. Key terms used throughout the document are defined. Five underlying *values* inform the SOBP: safety, quality, professionalism, accountability, and collaboration. Finally, we describe five *domains* of best practice: safe work environment; case development; SP training for role portrayal, feedback, and completion of assessment instruments; program management; and professional development. Each domain is divided into *principles* with accompanying key *practices* that provide clear and practical guidelines for achieving desired outcomes and creating simulations that are safe for all stakeholders. Failure to follow the ASPE SOBP could compromise the safety of participants and the effectiveness of a simulation session. Care has been taken to make these guidelines precise yet flexible enough to address the diversity of varying contexts of SP practice. As a living document, these SOBP will be reviewed and modified periodically under the direction of the ASPE Standards of Practice Committee as SP methodology grows and adapts to evolving simulation practices.

**Electronic supplementary material:**

The online version of this article (doi:10.1186/s41077-017-0043-4) contains supplementary material, which is available to authorized users.

## Introduction

Human simulation is a recognized methodology that involves human role players interacting with learners in a wide range of experiential learning and assessment contexts. At the inception of the practice, the human role players portrayed patients and were commonly referred to as standardized or simulated patients (SPs). In more recent years, SPs may portray an expanded scope of roles (e.g., clients, family members, healthcare professionals). There is increasing recognition that SP methodology can be applied to the work of any individual portraying a human in any simulation modality (e.g., confederates, learners playing roles other than themselves, technicians operating a manikin). At the same time, there also may be distinctions in the nature, scope, and function of those who portray roles. For example, confederates have been described as health professionals who are “planted” in a scenario to guide it while SPs act as a proxy for the person that they represent and often do not have a health professional background [1, 2].

The Association of Standardized Patient Educators (ASPE) is the global organization focused on human simulation [3]. ASPE’s mission is to share advances in SP-based pedagogy, assessment, research, and scholarship. It also supports the professional development of those who engage in human simulation. Therefore, it is incumbent on ASPE to pronounce underlying values and to establish Standards of Best Practice (SOBP) that ensure the growth and integrity of SP-based endeavors.

The ASPE SOBP provide clear and practical guidelines for educators who work with SPs. Care has been taken to make these guidelines precise and yet flexible enough to address the diversity of varying contexts of SP practice. Broader simulation practices are addressed in the International Nursing Association for Clinical Simulation and Learning (INACSL) Standards of Best Practice: Simulation^SM^ [4–11]. The ASPE SOBP are intended to be used in conjunction with the INACSL standards. Potential consequences of not following the ASPE SOBP relate to compromising both the safety of participants and the effectiveness of a simulation session.

## Process of SOBP development

The ASPE SOBP have been determined by the consensus opinion of a number of expert educators in the field of SP methodology. Experts have been identified as individuals who have contributed greatly to the scope and development of SP methodology, which had its inception in 1964. This consensus is based on evidence and practice, drawn from a variety of sources and methods, and reflects the perspectives of many cultures and fields of practice. In addition to citing specific references within this document, we also provide a list of essential references that informed its creation (Additional file 1: Essential Reading List).

The development of the standards began at a meeting (December, 2013) of a group of North American experts in the field of SP methodology selected by then ASPE President, Gayle Gliva-McConvey, and ASPE Standards of Practice (SOP) Committee Chair, Wendy Gammon (Table 1). A modified Delphi process [12] was used to identify domains, which form the basis of this document. A draft of this first round was presented at the January, 2014, meeting of the ASPE Board of Directors. Round two involved widening the field to include ASPE experts outside North America to review the domains and their principles (Table 2). Round three involved a final separate consensus for unification of this document by a team of reviewers (June, 2016) drawn from the ASPE Board of Directors (Table 3). These experts made final revisions (including changing the draft’s title from SOP to SOBP) and prepared this manuscript.

**Table 1:** Working Committee, December 2013

Carrie Bohnert	USA	Chair, ASPE Educational Content Committee, 2013–2015
Gail Furman	USA	National Board of Medical Examiners, founding member of ASPE
Wendy Gammon	USA	Chair, ASPE Standards of Practice Committee, 2013–2014
Gayle Gliva-McConvey	USA	President, ASPE, 2012–2013
Nancy McNaughton	Canada	Chair, ASPE Grants and Research Committee, 2014–2015
Cate Nicholas	USA	Chair, ASPE Grants and Research Committee, 2012–2013
Tamara Owens	USA	President, ASPE, 2008–2009
Sydney Smee	Canada	Medical Council of Canada
Diana Tabak	Canada	Chair, ASPE Hybrid Special Interest Group

**Table 2:** Reviewers, January 2014**–**2015

Connie Coralli	USA	Chair, ASPE Educational Resources Committee, 2013–2015
Melih Elcin	Turkey	Member Liaison, ASPE, 2014–2015
Valerie Fulmer	USA	Chair, ASPE Publications Committee, 2014–2015
Carine Layat-Burn	Switzerland	Chair, ASPE International Committee, 2014–2015
Karen Lewis	USA	President, ASPE, 2014–2015
Lorraine Lyman	USA	Chair, ASPE Standards of Practice Committee, 2014–2016
Debra Nestel	Australia	Simulated Patient Network
Jan-Joost Rethans	Netherlands	Chair, ASPE International Committee, 2007–2008
Karen Reynolds	United Kingdom	Vice President for Operations, ASPE, 2014–2015
Cathy Smith	Canada	Chair, ASPE Conference Committee, 2013–2016
Amber Walton	USA	Vice President for Operations, ASPE, 2011–2013

**Table 3:** Final Working Group, 2016**–**2017

Carrie Bohnert	USA	Vice President for Operations, ASPE, 2016–2017
Henrike Hölzer	Germany	Chair, ASPE International Committee, 2016–2017
Karen Lewis	USA	Chair, ASPE Standards of Practice Committee, 2017–2018
Lorraine Lyman	USA	Chair, ASPE Standards of Practice Committee, 2014–2016
Cathy Smith	Canada	Chair, ASPE Conference Committee, 2013–2016
Tonya Thompson	USA	Chair, ASPE Grants and Research Committee, 2016–2017
Amelia Wallace	USA	Chair, ASPE Educational Content Committee, 2016–2017

## Terms related to SP methodology

For the purposes of this document, we will expand on some key terms relevant to SP methodology. Our understanding of these terms is aligned with the definitions in the Society for Simulation in Healthcare’s (SSH) Healthcare Simulation Dictionary [13] and the INACSL Standards of Best Practice: Simulation^SM^ Simulation Glossary [11] and, in some cases, reflects additional nuances that are emerging from our practices.

The terms *standardized patient* and *simulated patient* (SP) are often used interchangeably and refer to a person trained to portray a patient in realistic and repeatable ways. SPs interact with learners in experiential education and assessment contexts. *Learners*, depending on the context, are variously described as students, trainees, participants, examinees, or candidates. SPs can also provide feedback on learner performance from the perspective of the person they portray, which is unique to working with SPs. As noted in the rationale, SP-based education has grown in size and scope of practice to include many different roles. For this reason, the term *simulated participant* is being used as a more inclusive term to refer to all human role players in any simulation context. In this document, the term SP refers to all of these nuances.

The context in which SPs are working determines the degree of repeatability or *standardization* (consistency and accuracy) of their behavior, both within an individual SP’s performance and between SPs portraying the same role. This behavior can be seen as part of a continuum. On one end of the continuum, in high stakes assessment, SPs may be trained to behave in a highly repeatable or standardized manner in order to give each learner a fair and equal chance and are often referred to as *standardized* patients. It is important to note that in this context, SPs are individuals whose behavior has been standardized. In formative educational settings, where standardization may not play an important part of the session design, carefully trained SPs are able to respond with more authenticity and flexibility to the needs of individual learners and are referred to as simulated patients.

The term *actor* is sometimes used to refer to an SP. While both SPs and actors are performing roles, and acting practices and theories can inform SP work, the scope of what an SP and an actor does is very different. In general, actors are fulfilling the objectives of a playwright and/or a director and perform for the entertainment of an audience. In healthcare simulation, actors may be hired to perform in an educational activity; however, as SPs, they are doing something different from actors. They are part of an educational team, focused on fulfilling the learning objectives of a simulation activity in service to learners.

We use the term *client* to refer to individuals or groups who contract with an SP program for various activities. The term *SP educator* is used to refer to those who work to develop expertise in SP methodology and are responsible for training and/or administering SP-based simulation. Some may be trainers who exclusively work with SPs, while some may be faculty or healthcare professionals who work with SPs as part of their clinical and/or academic roles.

## Discussion

The SOBP are organized into five *domains*: *safe work environment*; *case development*; *SP training for role portrayal*, *feedback*, *and completion of assessment instruments*; *program management*; and *professional development*. Each domain is divided into *principles* with accompanying key *practices*. The practices are numbered for ease of reference. Not all practices are applicable to every situation, and the order in which the practices emerge may vary.

**Fig. 1:**
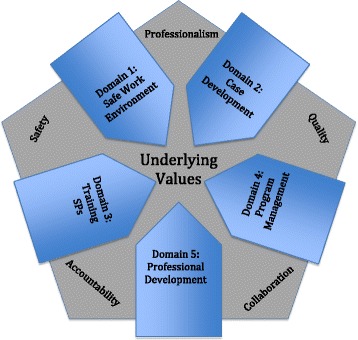
ASPE underlying values and SOBP domains

The domains are informed by five underlying *values* that support SP-based educational practices: *safety*, *quality*, *professionalism*, *accountability*, and *collaboration* (Fig. 1). Safety is the cornerstone of simulation practice. In that regard, it is the most central of all values because safety is a principle motivation for using simulation. In turn, simulation must be conducted in a safe manner that minimizes the risk to all stakeholders, no matter the activity. Quality refers to assuring and pursuing continuous improvement. We establish and follow standards of excellence in education, training, and research. Professionalism mandates that we are part of a community of professionals and act in accordance with common ethics, values, and standards. Accountability dictates a commitment to serving the needs of our stakeholders and informing the public about our practices. Collaboration requires sharing best practices with colleagues on a local and global scale. It is essential to the growth and development of SP-based practice.

While the domains and values are presented in separate sections, we acknowledge that they are not mutually exclusive. There are elements of all of the values in each of the domains, and there are overlapping practices that have been housed in each domain for ease of organization for the reader and to reiterate the importance of the practice (Fig. 1).

This foundational document offers both practical and at times, aspirational guidance. Future iterations of these standards will include more advanced and specialized domains, including SPs who train other SPs, facilitate sessions with learners, and act as teaching associates (e.g., in gynecological, male urogenital, and other physical examinations). This is a living document that will be reviewed and modified periodically under the direction of the ASPE Standards of Practice Committee as SP methodology grows and adapts to evolving simulation practices.

### Domain 1: safe work environment

It is incumbent on simulation educators to ensure that all stakeholders—be they SPs, learners, faculty, patients, or program staff—have a safe psychological and physical learning environment (see INACSL Standard: Professional Integrity [8]). For the community of SP educators, there are three distinct principles related to creating a safe work environment: safe work practices, confidentiality, and respect.

**Table:** 

**Principle**	**Practice**
1.1 Safe work practices	1.1.1 Ensure safe working conditions in the design of the activity (e.g., number of rotations, number of breaks, physical, cognitive, and psychological challenges in the role portrayal). 1.1.2 Anticipate and recognize potential occupational hazards, including threats to SP safety in the environment (e.g., allergenic substances, exposure to sharps, air quality, live defibrillators). 1.1.3 Screen SPs to ensure that they are appropriate for the role (e.g., no conflict of interest, no compromising of their psychological or physical safety). 1.1.4 Allow SPs to opt out of any given activity if they feel it is not appropriate for them to participate. 1.1.5 Brief SPs so they are clear about the guidelines and parameters of a simulation activity. 1.1.6 Provide SPs with strategies to mitigate potential adverse effects of role portrayal and prevent physical injury or fatigue. 1.1.7 Inform SPs and clients about the criteria and processes for terminating a simulation if they deem it harmful. 1.1.8 Structure time and create a process for de-roling and/or debriefing. 1.1.9 Monitor for and respond to SPs who have experienced adverse effects from participation in an activity. 1.1.10 Provide a process for SPs and clients to report adverse effects from participation in an SP activity (e.g., documentation and action steps to resolve the situation). 1.1.11 Support SPs who act in accordance with delineated program expectations if a complaint is made about them. 1.1.12 Manage client expectations of an SP’s possibilities and limitations. 1.1.13 Work with clients to clearly define the expected scope of SP involvement in work assignments.
1.2 Confidentiality	1.2.1 Understand the specific principles of confidentiality that apply to all aspects of each simulation event. 1.2.2 Ensure that SPs understand and maintain the principles of confidentiality related to specific simulation events. 1.2.3 Protect the privacy of the personal information of all stakeholders, including that which may be revealed within a simulation activity.
1.3 Respect	1.3.1 Respect SPs’ self-identified boundaries (e.g., modesty, limits to physical touch, impact on person). 1.3.2 Provide SPs with adequate information so that they can make informed decisions about participation in work assignments. 1.3.3 Ensure that SPs understand if and how they are being compensated before accepting work (e.g., may include payment for training and work time, travel expenses, food vouchers, gift cards).

### Domain 2: case development

While curricular or programmatic goals drive teaching and evaluation activities, the design and development of materials required for SP-based contributions to these activities are critical aspects of the SP educator role. For the purpose of this document, the materials include all descriptive case documents, any supporting documents (e.g., diagrams, photos, patient education literature, rating forms), evaluation instruments, training resources (e.g., references and videos), and training protocols an SP needs to prepare for a teaching or evaluation activity. It is important to recognize that SP cases have multiple components that reflect the different users of a case, such as SP educators, SPs, learners, raters, and administrators. The development of these materials is optimized when employing a collaborative, multistep process, utilizing a set of best practice guidelines for designing simulations (see INACSL Standard: Simulation Design [9]) as well as guidelines relevant to the professional context (e.g., medicine, law). Given the importance of case-related materials to the work of SPs, expertise in the development of teaching and evaluation materials is critical for SP educators. There are two principles that guide SP case development activities: preparation and case components.

**Table:** 

**Principle**	**Practice**
2.1 Preparation	2.1.1 Ensure that cases align with measurable learning objectives. (See INACSL Standard: Outcomes and Objectives.) 2.1.2 Identify and engage relevant subject matter experts to assist in the creation of materials. 2.1.3 Ensure that cases are based on authentic problems and respect the individuals represented in a case to avoid bias or stereotyping marginalized populations. 2.1.4 Ensure that case development process allows sufficient time to draft, review, and edit case materials prior to implementation. 2.1.5 Ensure that changes arising from dry-runs or other piloting processes are addressed prior to implementation of the case.
2.2 Case components	Ensure case components include the following when appropriate: 2.2.1 Clear goals and objectives that can be assessed. 2.2.2 Goals and objectives that specify the intended level of learners. 2.2.3 Simulation design that meets the purpose. 2.2.4 Simulation design that is repeatable. 2.2.5 Information for SPs (e.g., situation and backstory, history, affect and demeanor, signs and symptoms to simulate, cues). 2.2.6 Training resources (e.g., props, moulage, videos, task trainer). 2.2.7 Case-specific feedback or debriefing guidelines. 2.2.8 Briefing instructions, time frames, instructions to learners. 2.2.9 Evaluation instruments and performance measures (e.g., checklists and rating scales, participant and facilitator evaluations). 2.2.10 Training protocols for raters (SP or other). 2.2.11 Data for managing the documents and recruiting SPs (e.g., author information, date of development, patient demographics, body type criteria).

### Domain 3: SP training

SP training prepares SPs to portray roles, give feedback, and complete assessment instruments. These three areas are discrete skills, but are not mutually exclusive. It is the responsibility of the SP educator to integrate the development of these skills into SP training according to the learning objectives of the activity and the experience of the SP. Training can be done in many formats (e.g., face-to-face, online, blended).

The context in which SPs work determines the degree of standardization (consistency and accuracy) of their behavior, both within an individual SP’s performance and between SPs portraying the same role. SP educators apply the same training principles when preparing all simulated participants, including SPs, confederates, and others for all simulation modalities (e.g., hybrid, mixed-modality) [1, 2].

#### Role portrayal

SP educators are expected to ensure that SP performance is consistent and accurate. Because SPs are frequently asked to engage in roles that require at least a modicum of physical and emotional vulnerability, SP educators are required to provide supportive and safe training and learning environments (see the “Domain 1: safe work environment” section).

#### Feedback

Feedback is critical to learning. While learners may receive feedback from many educational sources, including clinicians and peers, SP feedback provides a unique perspective. As Berenson et al (2012) note: “SPs can provide students with unique and valuable information about how their actions and behaviors affected the SP’s emotional experience of the student, the SP’s trust in the student, and the SP’s understanding of the information exchanged. Thus, the SP’s feedback fills a critical educational role in the interpersonal and affective domains” ([14], pe-27). With appropriate training, SPs may also provide feedback on a learner’s communication, clinical, or procedural skills. Effective feedback requires knowledge of the models or protocols adopted by each institution, and SP educators may train SPs in oral and written feedback strategies.

#### Completion of assessment instruments

The Standards for Educational and Psychological Testing define assessment as “any systematic method of obtaining information from tests and other sources, used to draw inferences about characteristics of people, objects, or programs”([15],p72). In many assessment contexts, learners must demonstrate their competence through behavior that is assessed by observers. SPs often portray a role and observe behavior simultaneously. After an encounter, SPs may document learner performance on assessment instruments. If this is required, SP training must also focus on accurate and consistent completion of assessment instruments.

SP assessments may be formative, summative, or high stakes, can take many formats (e.g., single-encounter, multi-encounter, OSCE, CPX), and use many types of assessment tools (e.g., checklists, rubrics, narrative feedback). Expectations of SP performance vary, depending on the assessment type or format.

There are five principles SP educators should follow related to SP training methodology: preparation for the training process, training for role portrayal, feedback delivery, completion of assessment instruments, and reflection on the training process.

**Table:** 

**Principle**	**Practice**
3.1 Preparation for training	3.1.1 Review the purpose, objectives and outcomes (see INACSL Standard: Outcomes and Objectives), logistics, and case materials of the activity. 3.1.2 Address one’s own knowledge gaps, if any. 3.1.3 Create a training plan that is responsive to the context and format of each activity (e.g., group training for standardization, video review, practice with simulation equipment). 3.1.4 Gather training resources to supplement training. 3.1.5 Gather administration documents and special instructions.
3.2 Training for role portrayal	3.2.1 Review with SPs the key objectives, responsibilities, context (e.g., formative, summative, level of learner, placement in curriculum) and format (e.g., length of encounter, type of encounter) of each activity. 3.2.2 Engage SPs in discussion and practice of role portrayal features (e.g., affect, signs and symptoms, behaviors). 3.2.3 Provide SPs with strategies to deal with unanticipated learner questions and behaviors. 3.2.4 Ensure consistency and accuracy of role portrayal of individual SPs, and among groups of SPs portraying the same role. 3.2.5 Ensure SP readiness for the simulation activity through repeated practice and targeted feedback.
3.3 Training for feedback	3.3.1 Review with SPs the fundamental principles of feedback as they relate to the planned activity. 3.3.2 Inform SPs of the feedback objectives and level of the learners with whom they will be learning. 3.3.3 Inform SPs of the feedback logistics and setting (e.g., one-on-one feedback with learner, small group feedback, simulation debrief). 3.3.4 Train SPs to use their observations, responses, and knowledge to provide feedback on observable, modifiable behaviors in learners. 3.3.5 Ensure SP readiness through repeated practice and targeted feedback.
3.4 Training for completion of assessment instruments	3.4.1 Ensure that SPs understand the nature, context, and objectives of the assessment. 3.4.2 Ensure that SPs understand the format of the assessment instrument. 3.4.3 Ensure that SPs are able to complete assessment instruments in the time allotted. 3.4.4 Provide SPs with practice completing assessment instruments with a variety of learner behaviors. 3.4.5 Ensure that SPs understand both the principle and receptive experience of any physical exam maneuvers they will be assessing. 3.4.6 In formative assessment, ensure consistent and accurate completion of an assessment instrument within individual SPs, and among groups of SPs performing the same task. 3.4.7 In high stakes assessment, verify inter-rater reliability, in which a learner would achieve the same score when rated by different SPs. 3.4.8 In high stakes assessment, verify intra-rater reliability, in which SPs would assign the same score to an identical performance at different points in time.
3.5 Reflection on the training process	3.5.1 Reflect on one’s own training practices for future improvement (e.g., evaluation forms, debriefing, video review). (See also Domain 4.6: quality management.)

### Domain 4: program management

SP programs provide a trained cohort of SPs, expertise in SP methodology, and processes that administer SP services efficiently and cost effectively. Management in SP programs exists along a spectrum. Some programs may have one person dedicated to SP program administration and a few SPs, while others may be headed by a dedicated manager who oversees the work of many SPs, educators, and administrators. Regardless of size, SP programs are responsible for quality management practices, including quality planning, quality assurance, quality control, and quality improvement (see INACSL Standard: Professional Integrity [8]). Clearly stated policies and procedures allow an SP program to demonstrate that it meets legislated, institutional, and practice standards. They also specify approaches to meeting program goals, enable accountability to stakeholders (SPs, learners, faculty, staff), and encourage continuous improvement. There are six principles to address when managing SP programs.

**Table:** 

**Principle**	**Practice**
4.1 Purpose	4.1.1 Articulate a mission statement for the program. 4.1.2 Develop program goals. 4.1.3 Identify measurable objectives for each goal (where appropriate).
4.2 Expertise	4.2.1 Possess depth of knowledge in SP methodology. 4.2.2 Advocate for the integration of SP methodology into the curriculum where appropriate. 4.2.3 Identify when SPs should be incorporated into a simulation activity. 4.2.4 Collaborate with subject matter experts to design SP cases, training, and assessment materials. 4.2.5 Train SPs according to scenario or project parameters.
4.3 Policies and procedures	4.3.1 Develop and document policies to guide program activities. 4.3.2 Develop and document policies that take into consideration disability access and inclusion. 4.3.3 Develop and document business processes and procedures, including but not limited to creating financial management, business, and strategic plans. 4.3.4 Ensure policies and procedures are kept current and accessible. 4.3.5 Distribute policies and procedures to relevant stakeholders.
4.4 Records management	4.4.1 Collaborate with subject matter experts to develop a system for reporting learner performance to stakeholders (e.g., learners, curriculum developers, faculty, administration). 4.4.2 Ensure that policies are in place for case sharing and archiving. 4.4.3 Develop and document methods for securely storing, archiving, and destroying confidential data (e.g., SP records, learner data, video data, consent forms, release forms).
4.5 Team management	4.5.1 Consult with legal, financial, and human resources experts to ensure that status of SPs (e.g.. employee, independent contractor, volunteer) and compensation structure (if applicable) comply with institutional requirements. 4.5.2 Develop processes to identify, screen, interview, select, debrief, and maintain SPs and staff. 4.5.3 Recruit and maintain a cohort of SPs that reflects the diversity of the people they represent in simulation activities. 4.5.4 Establish policies and procedures for the psychological, physical, and environmental safety of SPs, learners, staff, and faculty. (See the “Domain 1: safe work practices” section.) 4.5.5 Advocate for ongoing professional development opportunities for all staff, including SPs.
4.6 Quality management	4.6.1 Gather data regularly to assess the alignment of program activities with legislated, institutional, and program policies and procedures. 4.6.2 Gather feedback regularly from SPs, learners, faculty, and other users regarding the quality of services provided by the program. 4.6.3 Analyze data and other feedback in a timely manner. 4.6.4 Implement changes for continuous improvement. 4.6.5 Inform stakeholders of changes made based on their feedback.

### Domain 5: professional development

SP educators engage in professional development to promote excellence in their own practices, within the community of practice, and among stakeholders. Professionalism has been defined for many professions that SP educators interact with, including medicine [16] and nursing [8, 17]. There are intersections with some of these concepts of professionalism. However, we are an emerging, heterogeneous practice without a licensing process. These SOBP are our first cohesive attempt to articulate the standards of professionalism for our practice. We draw on Steinert’s [18] model of faculty development to articulate professionalism and professional development as it relates to our context. In particular, we focus on three principles: career development, scholarship, and leadership.

**Table:** 

**Principle**	**Practice**
5.1 Career development	5.1.1 Develop and promote expertise in knowledge, skills, and attitudes related to SP-based simulation. 5.1.2 Develop and promote expertise in theories, principles, and processes of education and assessment relevant to the context of one’s practice (e.g., medical education, nursing education, legal, and law enforcement training). 5.1.3 Maintain membership in professional simulation societies (e.g., ASPE, ASPiH, INACSL, SESAM, SSH). 5.1.4 Engage in educational opportunities (e.g., professional conferences, courses, degree programs, certifications). 5.1.5 Develop personal management skills (e.g., time management, wellness strategies, career planning). 5.1.6 Seek out opportunities for career mentoring.
5.2 Scholarship	5.2.1 Develop an understanding of the range of opportunities for scholarship in SP methodology. 5.2.2 Identify and/or develop new contexts for SP methodology. 5.2.3 Contribute to the evolution of best practices through innovation, research, and dissemination of emerging methods in various venues e.g., publications, presentations).
5.3 Leadership	5.3.1 Promote understanding and development of SP methodology locally, nationally, and internationally. 5.3.2 Mentor and support SPs and other SP educators within one’s institution and within the community of practice. 5.3.3 Seek out and advocate for growth of leadership skills (e.g., collaboration, team building, change management, interpersonal effectiveness, conflict resolution).

## Additional file

Additional file 1: Essential Reading List. (DOC 32 kb)

## Abbreviations

ASPE: Association of Standardized Patient Educators
ASPiH: Association for Simulated Practice in Healthcare
INACSL: International Nursing Association for Clinical Simulation and Learning
SESAM: Society in Europe for Simulation Applied to Medicine
SOBP: Standards of Best Practice
SOP: Standards of Practice
SP: Simulated participant
SSH: Society for Simulation in Healthcare
