# Supplementary material for: The Association of Standardized Patient Educators (ASPE) Standards of Best Practice (SOBP)
Source: Adv Simul (Lond). 2017 Jun 27;2:10. doi: 10.1186/s41077-017-0043-4 (PMC5806371; doi:10.1186/s41077-017-0043-4)
Supplement: Supplementary file 1 — Essential Reading List. (DOC 32 kb) [file 41077_2017_43_MOESM1_ESM.doc]

# Additional file 1

## Essential Reading List
